# Single HA2 Mutation Increases the Infectivity and Immunogenicity of a Live Attenuated H5N1 Intranasal Influenza Vaccine Candidate Lacking NS1

**DOI:** 10.1371/journal.pone.0018577

**Published:** 2011-04-07

**Authors:** Brigitte M. Krenn, Andrej Egorov, Ekaterina Romanovskaya-Romanko, Markus Wolschek, Sabine Nakowitsch, Tanja Ruthsatz, Bettina Kiefmann, Alexander Morokutti, Johannes Humer, Janina Geiler, Jindrich Cinatl, Martin Michaelis, Nina Wressnigg, Sanda Sturlan, Boris Ferko, Oleg V. Batishchev, Andrey V. Indenbom, Rong Zhu, Markus Kastner, Peter Hinterdorfer, Oleg Kiselev, Thomas Muster, Julia Romanova

**Affiliations:** 1 Avir Green Hills Biotechnology AG, Vienna, Austria; 2 Influenza Research Institute, Russian Academy of Medical Sciences, St. Petersburg, Russia; 3 A.N. Frumkin Institute of Physical Chemistry and Electrochemistry of Russian Academy of Sciences (RAS), Moscow, Russia; 4 Christian Doppler Laboratory of Nanoscopic Methods in Biophysics, Institute for Biophysics, Johannes Kepler University Linz, Linz, Austria; 5 Institute for Medical Virology, Johann Wolfgang Goethe University, Frankfurt, Germany; Johns Hopkins University - Bloomberg School of Public Health, United States of America

## Abstract

**Background:**

H5N1 influenza vaccines, including live intranasal, appear to be relatively less immunogenic compared to seasonal analogs. The main influenza virus surface glycoprotein hemagglutinin (HA) of highly pathogenic avian influenza viruses (HPAIV) was shown to be more susceptible to acidic pH treatment than that of human or low pathogenic avian influenza viruses. The acidification machinery of the human nasal passageway in response to different irritation factors starts to release protons acidifying the mucosal surface (down to pH of 5.2). We hypothesized that the sensitivity of H5 HA to the acidic environment might be the reason for the low infectivity and immunogenicity of intranasal H5N1 vaccines for mammals.

**Methodology/Principal Findings:**

We demonstrate that original human influenza viruses infect primary human nasal epithelial cells at acidic pH (down to 5.4), whereas H5N1 HPAIVs lose infectivity at pH≤5.6. The HA of A/Vietnam/1203/04 was modified by introducing the single substitution HA2 58K→I, decreasing the pH of the HA conformational change. The H5N1 reassortants containing the indicated mutation displayed an increased resistance to acidic pH and high temperature treatment compared to those lacking modification. The mutation ensured a higher viral uptake as shown by immunohistochemistry in the respiratory tract of mice and 25 times lower mouse infectious dose_50_. Moreover, the reassortants keeping 58K→I mutation designed as a live attenuated vaccine candidate lacking an NS1 gene induced superior systemic and local antibody response after the intranasal immunization of mice.

**Conclusion/Significance:**

Our finding suggests that an efficient intranasal vaccination with a live attenuated H5N1 virus may require a certain level of pH and temperature stability of HA in order to achieve an optimal virus uptake by the nasal epithelial cells and induce a sufficient immune response. The pH of the activation of the H5 HA protein may play a substantial role in the infectivity of HPAIVs for mammals.

## Introduction

An unprecedented spread of highly pathogenic avian influenza viruses (HPAIV) of the H5N1 subtype was observed among wild and domestic birds throughout the last decade. Hundreds of cases of the direct transmission of avian viruses to humans with a case fatality rate exceeding 50% raised great concerns of a possible new pandemic.

Numerous clinical studies with vaccines produced from H5N1 viruses have demonstrated that the inactivated vaccines produced from the H5 hemagglutinin (HA) appeared to be poorly immunogenic compared to seasonal influenza strains [1], [2]. A broader and longer lasting immunity might be induced by live attenuated influenza vaccines, which are believed to be superior to inactivated vaccines [3], [4]. However, H5N1 cold adapted vaccine strains comprising surface antigens derived from A/Vietnam/1203/04 (VN1203) or A/Hong Kong/213/03 lacked replication in the human nasal mucosa, correlating with the observed poor immunological outcome [5].

The effectiveness of intranasal live attenuated influenza vaccines is substantially dependent on the efficient virus uptake and subsequent replication in the cells of the upper respiratory tract. Human influenza viruses are known to attach predominantly to the surface of ciliated epithelial cells in the human trachea, bronchi, and bronchioles while avian H5N1 viruses prefer the lower respiratory tract, in turn binding more abundantly to the alveoli [6]. This could be explained by the preferential affinity of H5 HA to sialic acid receptors with an α2,3 galactose (α2,3Gal) linkage dominating on the cells of the lower respiratory tract, but not to the α2,6Gal type, which is abundantly present in the human trachea [7], [8]. However, in spite of the difference in the receptor specificity, it was demonstrated that H5N1 viruses are able to infect *ex vivo* cultures of human nasopharyngeal, adenoid, and tonsillar tissues [9]. Consistently, another live attenuated H5 vaccine candidate comprising the HA of the low pathogenic avian influenza virus (LPAIV) A/duck/Potsdam/86/92 (H5N3) was shown to replicate efficiently in the human upper respiratory tract (for at least 11 days) [10], [11]. Therefore, the receptor specificity properties of influenza surface glycoprotein might not be the only responsible reason for the low infectivity of avian viruses in humans.

Unlike isolated epithelial cells *in vitro*, the human airway luminal surface *in vivo* presents a significant extracellular barrier to influenza infection. It includes the mucociliary clearance system, viscous fluids, and macrophages interfering with the virus access to the cell surface. In addition, the measurement of the pH of the human nasal cavity revealed that the overall pH range at the anterior and posterior sites is 5.2–8.0 [12]–[15]. Acidic pH, heat, or chemical denaturants are known to promote the conformational change of HA into its fusogenic form, which is responsible for the complete viral inactivation [16]. Therefore, in order to overcome the human mucosal barrier, influenza viruses require a certain level of HA stability towards inactivating factors.

It was described that human and LPAIVs are relatively resistant to an acidic pH environment because they perform the pH dependent conformational change of the HA at a pH range of 5.1 to 5.4 [17]. In contrast, the HAs of H5 and H7 HPAIVs are much more sensitive undergoing conformational modification already at a pH of 5.6 to 6.0 [18]. By the mutagenesis of viral HA, Reed et al. succeeded in increasing the environmental stability of an HPAIV H5N1 strain (A/Chicken/Vietnam/C58/04) through the reduction of the pH of HA activation [19]. The obtained mutant demonstrated reduced virulence and transmissibility in wild ducks.

We hypothesized that the poor infectivity of HPAIVs for mammals and, as a consequence, the insufficient efficacy of live attenuated H5 vaccines is related to the low stability of their HA protein. In order to prove our hypothesis, we constructed two H5N1 live attenuated vaccine candidates lacking NS1 based on the VN1203 virus, which differ in a single amino acid substitution 58K→I in the HA2 subunit [20]. Reed et al. described this mutation as decreasing the threshold pH of the activation of the H5 HA protein of approx. 0.5 pH units [21]. The obtained viruses were compared for their stability *in vitro* and for the infectivity and immunogenicity after intranasal application *in vivo* in mice.

We demonstrated that the stabilization of the HA molecule towards acidic pH and high temperature promotes superior vaccine virus infectivity in the respiratory tract and, consequently, increased immunogenicity in mice.

## Results

### HPAIVs but not human seasonal viruses lose their infectious activity at an acidic pH in primary Human Nasal Epithelial cells (HNEpC)

**Figure 1:**
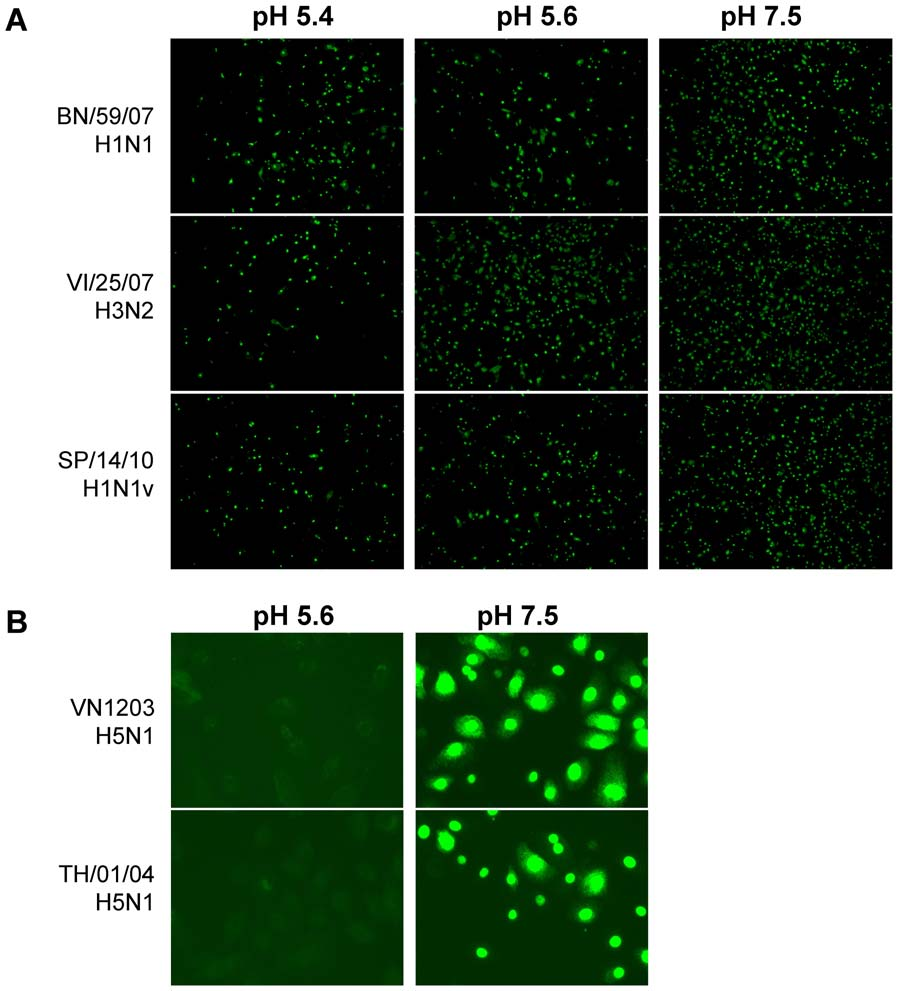
Infectivity of human and highly pathogenic avian viruses at an acidic pH in HNEpCs. Primary Human Nasal Epithelial cells were infected with human (A) epidemic BN/59/07 (H1N1), VI/25/07 (H3N2), SP/14/10 (H1N1v) or avian (B) highly pathogenic viruses VN/1203 (H5N1) and TH/01/04 (H5N1) at the indicated pH values. Influenza NP protein was visualized by immunostaining after incubating for 5 h.

The acidification machinery of the human nasal passageway is known to release protons in response to irritation or inflammation [22] acidifying the mucosal surface (down to pH of 5.2). Therefore, viruses must possess a certain level of stability to low pH in order to infect the upper respiratory tract. We analyzed whether human seasonal virus isolates differ from HPAIVs in infectious activity at an acidic pH. Human viruses A/Brisbane/59/2007 (H1N1) (BN/59/07), A/Vienna/25/07 (H3N2) (VI/25/07), A/St. Petersburg/14/10 (H1N1v) (SP/14/10) and HPAIVs VN1203 (H5N1) and A/Thailand/01/04 (H5N1) (TH/01/04) of an early passage level adjusted to a similar multiplicity of infection (moi) were admixed with acidic (pH 5.4 or/and 5.6) or neutral (pH 7.4) buffer followed by the inoculation of the HNEpCs. After removing the inoculum, the cells were incubated at a neutral pH for 5 hours without trypsin. The effectiveness of the infection was visualized by influenza nucleoprotein (NP) staining (Fig. 1). All human influenza viruses, including the recently appeared pandemic H1N1v virus, were capable of infecting cells at acidic conditions (Fig. 1A), while both highly pathogenic H5N1 viruses infected HNEpCs only at a neutral pH (Fig. 1B). A similar pH dependence of infection was observed in Vero cells (results are not shown).

### Generation of H5N1 viruses with 58K→I substitution in the HA2 subunit

To investigate whether the infectivity and, consequently, the immunogenicity of H5N1 viruses might be improved by decreasing the threshold pH of HA fusion to the range described for human influenza viruses, we modified the HA protein by implementing the 58K→I substitution in the HA2 subunit of the VN1203 virus, according to Reed et al. [21]. This mutation located in the HA2 coiled-coil domain was shown to decrease the pH of the activation of the H5 HA protein. An additional attempt failed to rescue a corresponding virus comprising HA2 23G→C modification, which is described to decrease the pH of the fusion of H7N7 viruses [23].

Increased resistance of influenza HA to an acidic pH is known to be linked to a decreased isoelectric point (pI) of the HA2 protein subunit, the pH value at which the protein charge is neutral [24]. The calculated value pI = 4.59 for the mutated HA2 subunit was lower than that of the natural VN1203 HA2 subunit (pI = 4.67), supporting the assumption of the enhanced resistance of the mutated HA to acidic pH (http://isoelectric.ovh.org/EMBOSS).

The reassortant virus containing mutation HA2 58K→I and the deletion of the NS1 ORF was generated by the eight-plasmid rescue method in Vero cells analogously to the previously described VN1203ΔNS1 virus [20] and was named VN1203ΔNS1-K58I. Both viruses comprised an HA with a polybasic cleavage site modified in a trypsin dependent manner [25]. In addition, a corresponding pair of reassortants with a complete NS segment was generated in the same way and named VN1203wtNS and VN1203wtNS-K58I, respectively.

### Mutation HA2 58K→I decreases the pH threshold of HA fusion and increases the virus resistance to acidic pH and high temperature

**Figure 2:**
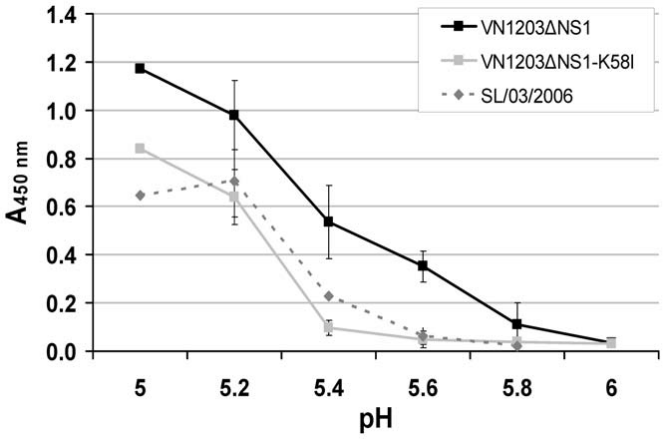
Hemolytic activity of influenza viruses as a function of pH. Viruses VN1203ΔNS1, VN1203ΔNS1-K58I, and seasonal A/SL/03/06 (H1N1) standardized to 128 HA units were mixed with 1% suspension of human erythrocytes at 0°C and pH 7.4 for 1 h and then the buffer was replaced with MES buffer at various pH values. After incubation at 37°C for 1 h hemolysis representing the fusion activity was determined spectrophotometrically at 405 nm. Error bars represent the standard deviation from triplicate experiments.

To prove the impact of the introduced mutation on the pH optimum of HA fusion activity, we analyzed the reassortants VN1203ΔNS1 and VN1203ΔNS1-K58I in a fusion assay. The original virus had the capacity to trigger a fusion at pH values≤5.6, whereas the same fusion rate for the mutant virus was observed at pH≤5.3 (Fig. 2). It is noteworthy that the mutant H5N1 virus VN1203ΔNS1-K58I showed the same activation profile of HA as observed for the human seasonal virus A/Solomon Island/03/06 (H1N1) (SL/03/06).

The result of the HA fusion assay was confirmed by the atomic force microscopy (AFM) technique allowing visualization of viral interaction with the lipid membrane. Unlike the electron microscopy, this technique enables the imaging of biological samples in a natural fluid environment with atomic resolution. Virus particles were adsorbed on a supported bilayer lipid membrane (sBLM) containing GD1α ganglioside, which served as a receptor for the influenza virus. The conversion of the conformation of HA at the threshold pH value leading to the activation of the fusion of the virus envelope with sBLM was registered by changing the domain structure of the lipid bilayer. We found that the HA conformational change of VN1203ΔNS1 occurred already at pH 5.8, whereas the VN1203ΔNS1-K58I mutant was stable under these conditions. The image confirming the conformational change of the mutant virus was observed at pH 5.0 (detailed data are presented as supporting information, Text S1, Fig.S1).

**Figure 3:**
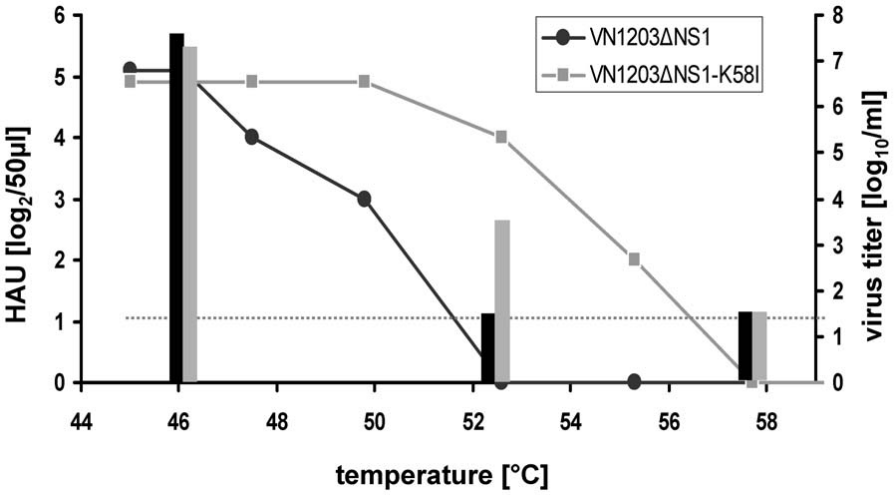
Effect of elevated temperature on the viral HA and infectious titer. Virus preparations were incubated at indicated temperatures for 30 min. HA titers (bars, left Y-axis) and infectious titers measured as log_10_ TCID_50_ (graphs, right Y-axis) were determined for VN1203ΔNS1 (black bars and lines) and VN1203ΔNS1-K58I (gray bars and lines) viruses. The lower limit of detection for the TCID_50_ titer is 1.5 log_10_ TCID_50_/ml indicated by the horizontal dashed line.

Next, we investigated whether the virus VN1203ΔNS1-K58I, which was more resistant to an acidic pH, was also more stable at an increased temperature. VN1203ΔNS1 and VN1203ΔNS1-K58I viruses were incubated at a temperature ranging from 46°C to 58°C for 30 min with the subsequent measurement of the remaining infectious and hemagglutination titers. We found that the infectivity of the VN1203ΔNS1 virus was impaired already at 50°C, as indicated by a drop in the infectious titer more than 100 times. At the same time, this temperature did not affect the infectious titer of the mutant virus (Fig. 3). After incubation at 52°C, the VN1203ΔNS1 virus completely lost the infectious titer, whereas the titer of the mutant virus VN1203ΔNS1-K58I decreased by 1.0 log_10_ TCID_50_/ml. Analogous results were obtained by assessing HA titers indicating improved temperature stability of mutated surface glycoprotein.

### Mutation HA2 58K→I preserves virus infectivity at an acidic pH in cell culture

**Figure 4:**
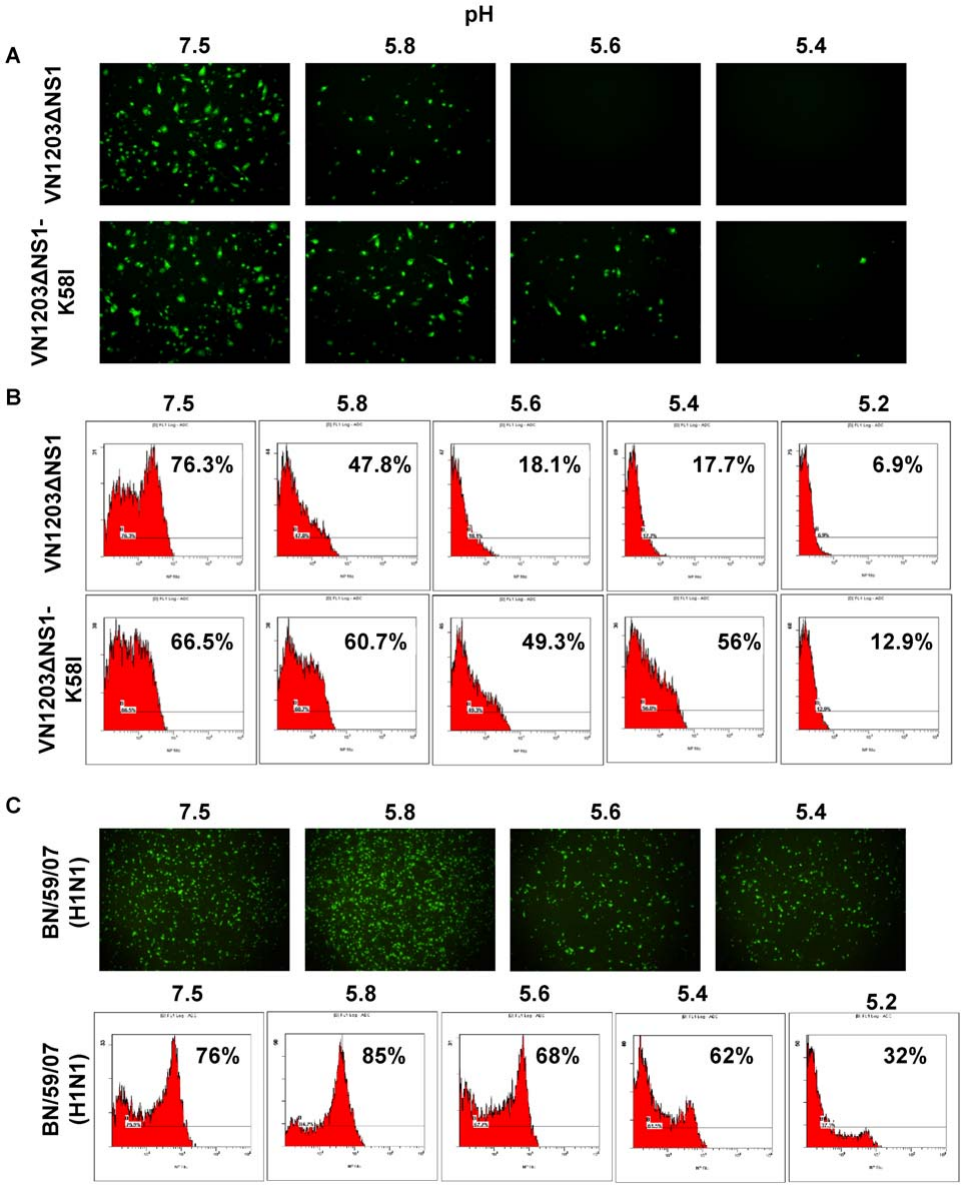
Infectivity of viruses at different pH values in tissue culture. (A) Virus VN1203ΔNS1 or VN1203ΔNS1-K58I was mixed with MES buffer at indicated pH values and used for infection of Vero cells at moi 2. Influenza NP was visualized by immunostaining after incubating for 5 h. (B) MDCK cells were infected with VN1203ΔNS1 or VN1203ΔNS1-K58I viruses at indicated pH values. The number of infected cells was determined after the immunostaining of viral NP (5 h p.i.) by flow cytometry. The indicated numbers show the percentage of infected cells. (C) Vero or MDCK cells were infected with seasonal human influenza virus BN/59/07 (H1N1) at indicated pH values. Virus infectivity was analyzed analogously by immunostaining or by flow cytometry.

The infectivity of reassortants VN1203ΔNS1 and VN1203ΔNS1-K58I under acidic conditions was compared in Vero cells at pH 5.4, 5.6, 5.8, or 7.4. The human BN/59/07 virus was taken as a control sample resembling a pH stable phenotype. As is shown in Fig. 4A, the mutant virus VN1203ΔNS1-K58I gained a resistance to low pH infecting Vero cells at pH 5.6, whereas the limit of infection was at pH 5.8 for the original virus VN1203ΔNS1. These results were confirmed on MDCK cells estimated by the flow cytometry analysis under similar conditions (Fig. 4B). As expected, the human BN/59/07 (H1N1) virus was infectious on Vero cells at pH 5.4 and infected 32% of the MDCK cells even at pH 5.2 (Fig. 4C).

### Mutation HA2 58K→I increases virus sensitivity to lysosomotropic agents

**Figure 5:**
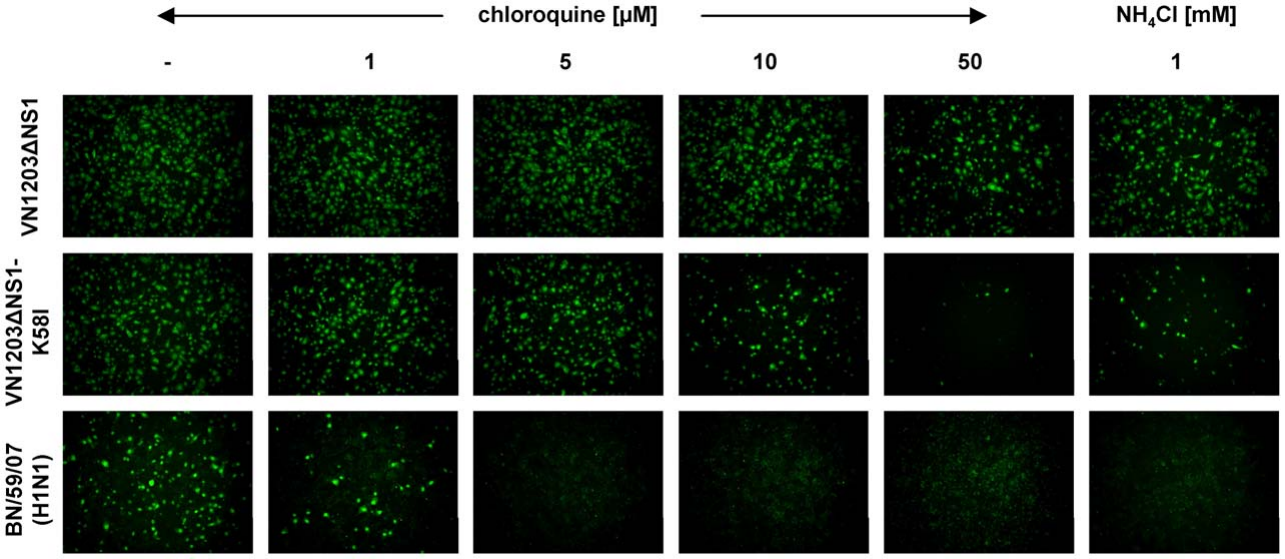
Viral sensitivity to the lysosomotropic agents chloroquine or NH_4_Cl. Pretreated Vero cells were infected with VN1203ΔNS1, VN1203ΔNS1-K58I, or seasonal BN/59/07 (H1N1) virus at moi 5 in a medium supplemented with chloroquine or NH_4_Cl at indicated concentrations. The infected cells were visualized by the immunostaining of influenza NP (5 h p.i.).

Avian influenza viruses in contrast to human strains are more resistant to the prevention of endosomal acidification mediated by lysosomotropic agents, such as chloroquine and NH_4_Cl [24]. Therefore, we compared the infectivity of both viruses in the presence of chloroquine or NH_4_Cl (Fig. 5). As a control, the seasonal influenza strain BN/59/07 (H1N1) was included in the study. The infection of Vero cells with VN1203ΔNS1-K58I was decreased by 10 µM chloroquine and completely inhibited by 50 µM. In contrast, the growth of the original virus was hardly affected even at 50 µM chloroquine. BN/59/07 (H1N1) was most sensitive to chloroquine as the infection was reduced by 1 µM. These results were confirmed by another lysosomotropic agent (1 mM NH_4_Cl) when the infectivity of the VN1203ΔNS1-K58I or BN/59/07 (H1N1) viruses was almost lost, whereas the VN1203ΔNS1 virus was not affected. Thus, the mutation HA2 58K→I converted the phenotype of the avian influenza virus to be more similar to that of the human isolate.

### Mutation HA2 58K→I decreases the replication efficiency of H5N1 viruses in cell culture

**Figure 6:**
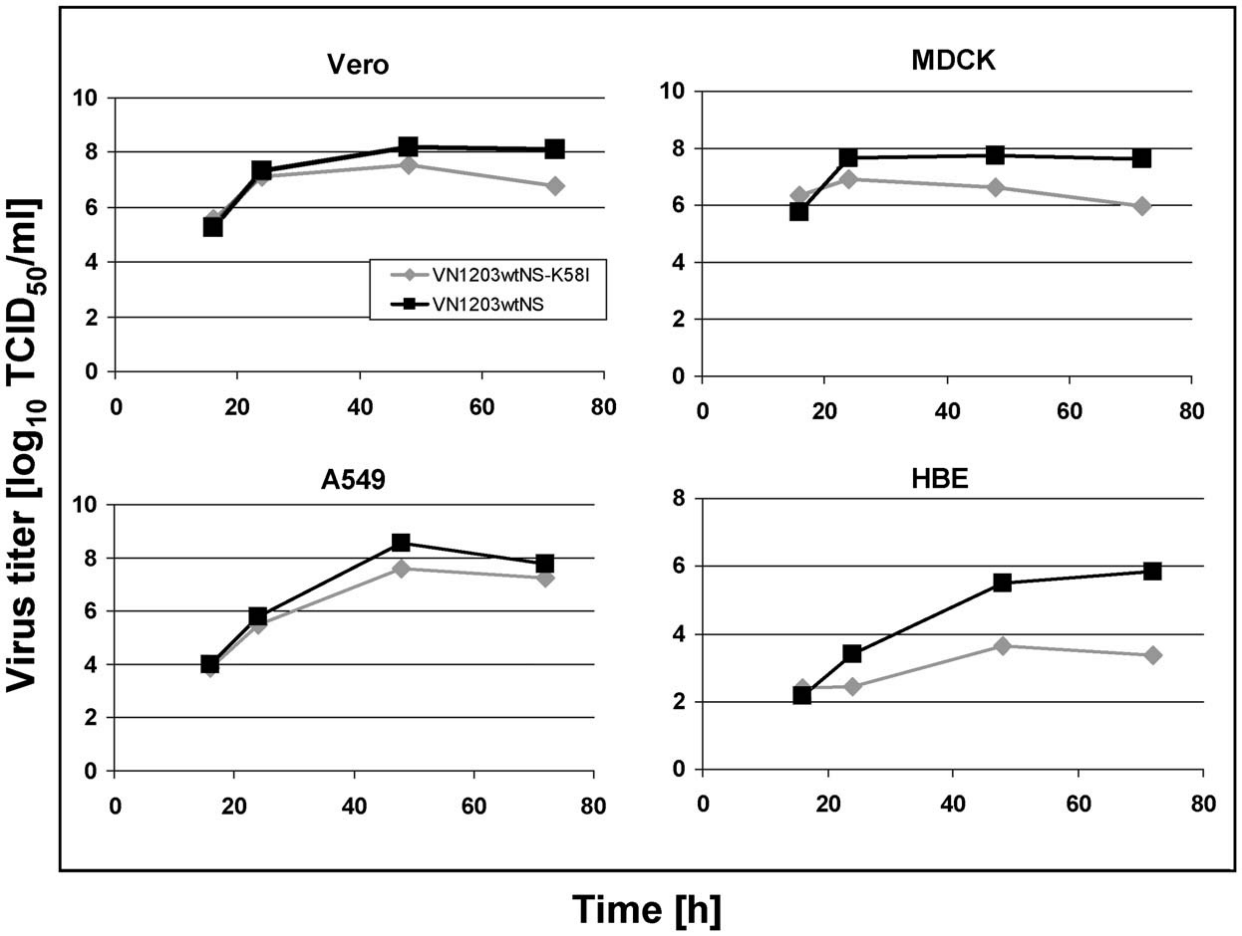
Growth curves on different cell lines. Vero (A), MDCK (B), A549 (C), or HBE (D) cells were infected with VN1203wtNS or VN1203wtNS-K58I virus at moi 0.001. Supernatants were collected at indicated time points and virus infectious titers were determined by TCID_50_ assay on Vero cells.

To elucidate whether the changed stability of HA to an acidic pH has any influence on the replication efficiency in various cell lines, we compared the viral growth capacity in Vero, MDCK, A549 (human lung carcinoma cell), and in HBE (human bronchial epithelial) cells. As the ΔNS1 viruses are replication deficient in interferon competent cell lines (all mentioned above except Vero), two similar viruses expressing functional NS1 protein VN1203wtNS and VN1203wtNS-K58I were used in these experiments. Similar results of stability towards an acidic pH and elevated temperature were obtained for these viruses (results are not shown). Growth curves made for each cell line revealed that the introduced mutation HA2 58K→I impaired the yield of the mutant virus VN1203wtNS-K58I in HBE cells of about 150 times. A similar tendency was observed in Vero, MDCK, and A549 cells (Fig. 6). Therefore, a mutation decreasing the pH threshold of the HA conformational change may affect the growth of H5N1 viruses in continuous cell lines.

### Mutation HA2 58K→I increases virus infectivity in mice

**Figure 7:**
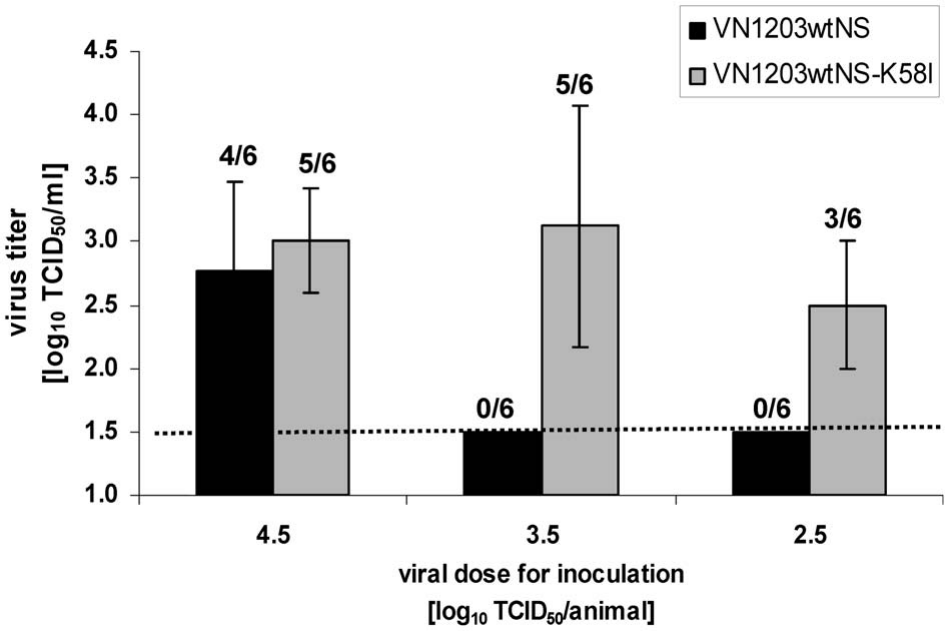
Viral growth capacity in the mouse upper respiratory tract. Mice were infected i.n. at doses of 2.5, 3.5, or 4.5 log_10_ TCID_50_/animal of VN1203wtNS or VN1203wtNS-K58I virus. The presence of the virus was determined in 10% w/v nasal tissue homogenates by TCID_50_ assay 60 h p.i. The lower limit of detection for the TCID_50_ titer is 1.5 log_10_ TCID_50_/ml indicated by the horizontal dashed line. The number of animals infected/total number is indicated above the bars. Error bars represent the standard deviation.

To assess the relevance of the *in vitro* findings in animals, we compared the virus infectivity in mice. First, we measured the MID_50_ of viruses with mutant or original HA. The animals were infected with VN1203wtNS or VN1203wtNS-K58I viruses in a dose ranging from 2.5 to 4.5 log_10_ TCID_50_/animal. Nasal tissues were collected 60 h after i.n. administration and the tissue homogenates were investigated for the presence of infectious virus by TCID_50_ assay. The mutant virus VN1203wtNS-K58I had an approximately 25-fold lower MID_50_ (MID_50_ 2.9 log_10_) compared to that found for the original virus (MID_50_ 4.3 log_10_) (Fig. 7). This result was consistent in two independent experiments.

**Figure 8:**
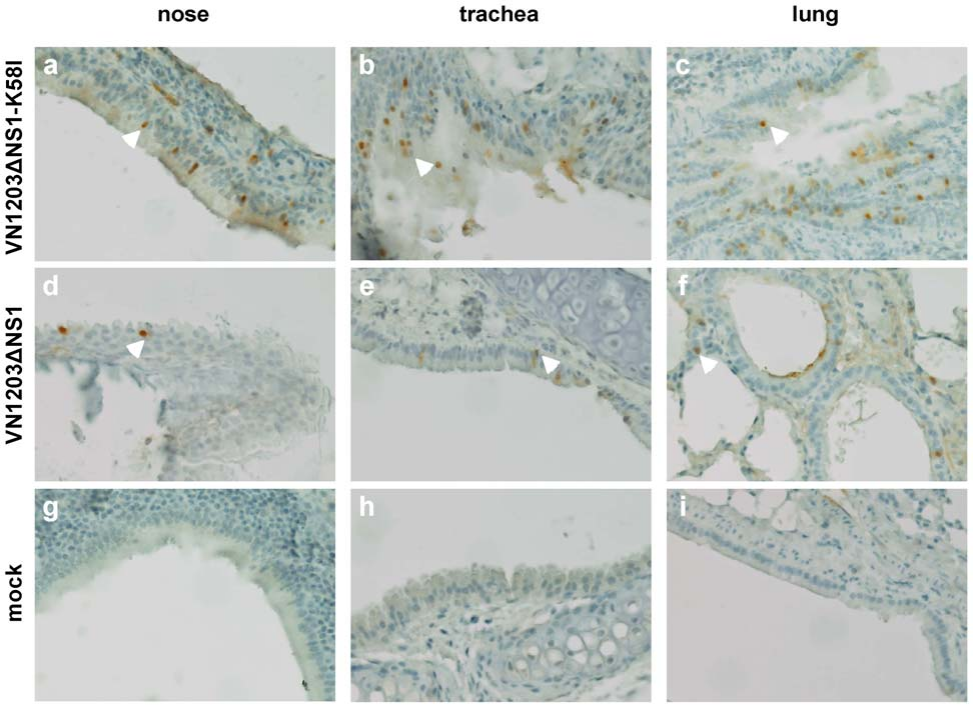
Viral infectivity in the mouse respiratory tract. Mice were infected i.n. with 6 log_10_ TCID_50_/animal of VN1203ΔNS1 or VN1203ΔNS1-K58I virus or mock-infected with PBS. Nasal, tracheal, and lung tissues were examined 12 h p.i. for the presence of virus infected cells by the immunostaining of influenza NP.

To compare the extent of the uptake of the original and mutated viruses, the animals were infected i.n. with live attenuated replication deficient VN1203ΔNS1 or VN1203ΔNS1-K58I vaccine candidates to ensure single round infection at a dose of 6 log_10_ TCID_50_/animal. A qualitative assessment of infected foci was carried out by the immunohistochemistry of the sections obtained from the nasal mucosa, trachea, and lung tissues stained with anti-influenza NP primary antibody. The mutant VN1203ΔNS1-K58I virus increased the extent of the infection of the alveolar regions of lungs, trachea and nasal mucosa, where more foci of the infection were seen for all 5 immunized animals when compared to the original VN1203ΔNS1 virus (Fig. 8). Thus, the introduced mutation promoted more intensive viral uptake in the mouse respiratory tract.

### Mutation HA2 58K→I contributes to increased virus immunogenicity in mice

**Figure 9:**
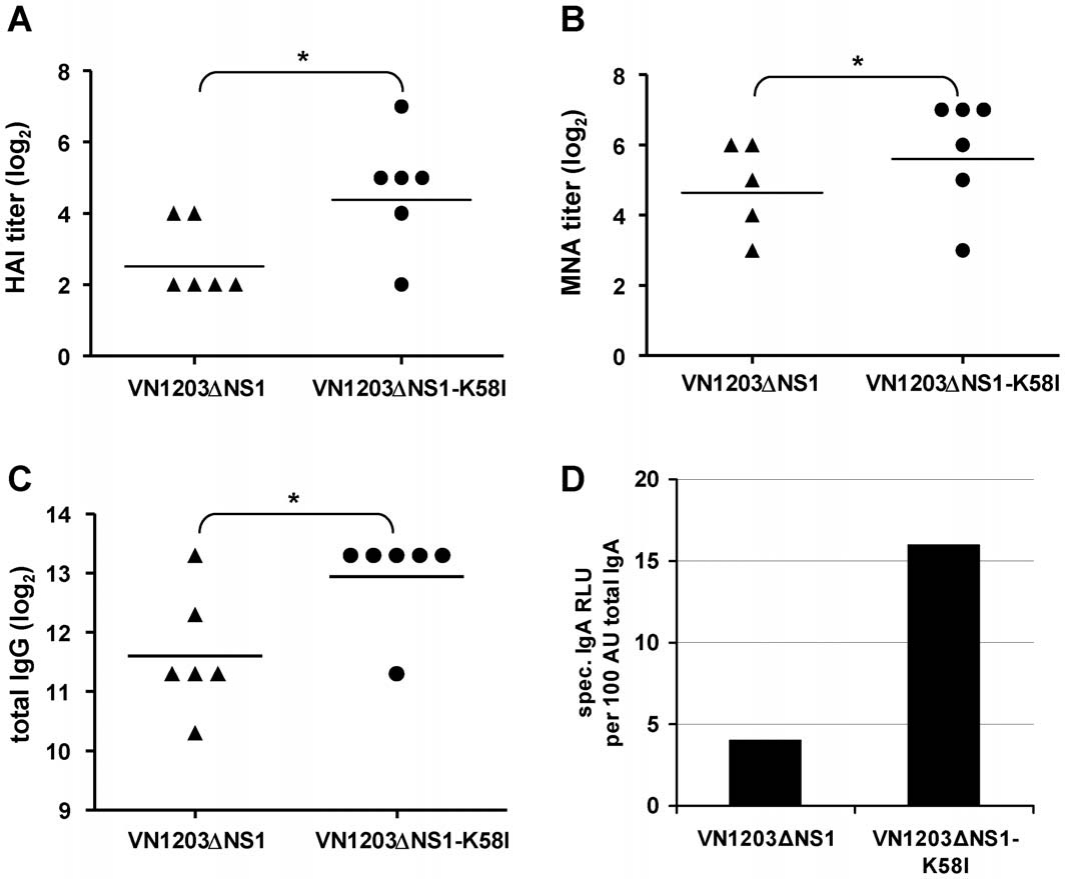
Antibody titers after single immunization of mice. Mice were vaccinated i.n. with 6 log_10_ TCID_50_/animal of VN1203ΔNS1 or VN1203ΔNS1-K58I virus. Sera (collected 4 weeks p.i.) were analyzed for HAI (A), neutralizing (B) and for total IgG (C) antibodies measured by ELISA. The titers of the individual animals (symbols) and the geometric mean titers (horizontal lines) are indicated. An undetectable antibody titer was assigned a value of 4 (HAI; A) or 8 (MNA; B). (D) Pools of nasal washes (collected 4 weeks p.i.) were examined for the presence of homologous IgA antibodies. * indicates p<0.05 determined by a t-Students test.

In a previous study, we noticed that vaccine candidate VN1203ΔNS1 did not induce any HAI antibodies in mice in contrast to macaques and ferrets, although still providing protection against challenge viruses [20]. Therefore, we were interested in whether modified VN1203ΔNS1-K58I virus is able to evoke functional HAI or neutralizing antibodies in a mouse model. A single i.n. vaccination with the VN1203ΔNS1 virus induced detectable HAI titers only in 2 out of 6 animals (GMT = 6.3), whereas the administration of the mutant virus VN1203ΔNS1-K58I induced a response in 5 animals (GMT = 25.4) (Fig. 9A). An MNA test also revealed higher titers of neutralizing antibodies induced by VN1203ΔNS1-K58I (GMT = 57) virus than that induced by VN1203ΔNS1 (GMT = 27.9) (Fig. 9B). The significant superior induction of specific antibodies by the mutated vaccine virus was confirmed in an ELISA, where the IgG GMT evoked by VN1203ΔNS1 was 3225 and by VN1203ΔNS1-K58I was 8127 (Fig. 9C). The measurement of specific IgA antibodies in pooled nasal washes of immunized mice indicated fourfold more antibodies after immunization with the mutant virus (Fig. 9D). Thus, a single immunization of mice with the VN1203ΔNS1-K58I virus, containing the stability improved HA, induced a significantly higher immune response as reflected by increased serum and mucosal antibody titers.

Despite the observed differences in immunogenicity, both VN1203ΔNS1 and VN1203ΔNS1-K58I viruses completely prevented the replication of the homologous challenge virus VN1203wtNS in the lungs of 3/5 and 4/5 animals, respectively, taken 3 days post immunization, (data not shown) showing no statistically significant difference.

## Discussion

The effective spread of influenza viruses in the human population may require a certain level of environmental stability of the virus particles depending on a pathway of their transmission. Three modes of infection with influenza viruses in people have been postulated, including aerosol transmission, infection with large droplets, and self-inoculation of the nasal mucosa by contaminated hands [26]. Although, these routes of infection are not mutually exclusive, effective infection via the nasal route could be dependent on the pH stability of the virus particles due to the mechanisms of the fast acidification of human nasal mucosal surfaces [22].

Similar to the previous observation [27], we found that human epidemic influenza viruses, irrespective of their subtype (H1, H1v, H3), are more resistant to physical factors such as low pH (5.4–5.6) or elevated temperature exposure than highly pathogenic H5N1 influenza strains. It should be mentioned that the pH stability phenotype might be abrogated upon passaging in tissue culture [28]. Therefore, the virus isolates of the early passage level were used in this study. Contrary to human strains, HPAIVs and their derivates with a modified HA cleavage site were not infectious at pH≤5.6.

We hypothesize that this relatively low pH stability of HPAIVs viruses might be a limiting factor for infectivity in the mammalian upper respiratory tract particularly in the nasal compartment. To prove the hypothesis we introduced a known stabilizing mutation 58K→I into HA2 subunit of the VN1203 H5N1 virus and compared the properties of the obtained viruses *in vitro* and *in vivo*. We revealed that the *in vitro* parameters, such as the pH threshold of the HA conformational change, thermo-stability of HA protein, and virus sensitivity to drugs preventing the endosomal acidification of mutated virus, correspond (although not to the full extent) to those of the human influenza isolates. Moreover, by applying the FMA technique, we could reveal that the conformational change of the original, but not the mutated HA, occurs already at pH 5.8.

Interestingly, the introduction of the 58K→I substitution impaired the viral growth of the pH stable mutant in HBE cells. A similar finding was made for the HA2 G23→C mutant of A/Nederlands/219/03 (H7N2) virus with a decreased pH of HA activation showing a reduced replication in MDCK cells [23]. Thus, decreasing the pH value of HA activation to the level of human viruses might not be beneficial for growth in tissue culture. These data are consistent with the previous observation showing that the adaptation of human influenza viruses to high growth in MDCK cells was accompanied with the appearance of mutations that elevate the pH of HA membrane fusion [28].

In contrast to the tissue culture results, the intranasal infection of mice revealed that the pH stable variant was more infectious in the mouse respiratory tract, including nasal mucosa, trachea, and lungs, as demonstrated by immunohistochemistry. Such a difference could not be explained by the superior replication capacity of the mutant virus, since NS1 protein deletion abrogated the multicycle replication. [20], [29].

For an analogous pair of viruses comprising a functional NS1, the HA2 modification diminished the MID_50_ 25 times compared to the non-mutated strain. These results are in agreement with the data of Ilyushina et al., showing that equivalent modifications of the HA of an H7N7 virus decreased the mouse lethal dose 50 by 3 log_10_ units [23]. Reed et al. demonstrated, for a highly pathogenic H5N1 strain, that the reduction of the pH of HA activation increased the viral shedding from the trachea but not from the cloaca of infected birds, indicating that the threshold pH of HA conformational change could be important for host and/or tissue tropism [19]. Consistently for better viral uptake, the VN1203ΔNS1-K58I virus appeared to be more immunogenic in mice, inducing significantly higher HAI and neutralizing antibody titers as well as mucosal IgA antibodies. We observed a similar regularity for seasonal influenza viruses in ferrets where the stable virus induced an enhanced antibody response when compared to the mutant having HA destabilizing mutation [30].

The acidification of the mucosal surfaces is one of the oldest primary innate defense mechanisms in mammals against a variety of pathogens. The degree of acidification depends on the distribution of the submucosal glands responsible for the liquid secretion and, therefore, might differ in various animal species [31]. There is evidence that mouse epithelium cell composition and ion transport traits are similar to that of the human conducting airways [32], [33]. The nasal epithelia of humans and mice are abundantly supplied with qualitatively similar glands [34]. The pH of the human nasal mucosa in healthy adults is known to be slightly acidic [12]–[15]. Moreover, the human nasal airway epithelium starts to release acid in reaction to organic dust or inflammation [22], [34]. In combination with the receptor specificity factor, the human nasal compartment might be suboptimal for an efficient infection by HPAI viruses, hence requiring a low respiratory tract delivery by aerosols (reviewed in [26]). In this regard, mutations decreasing the pH of HA conformational change and, therefore, stabilizing the HA of H5 HPAIVs might be a prerequisite for their efficient spread in humans via a large droplet mechanism infecting the upper respiratory tract (nose). A relatively high pH of HA activation (ranging from 5.6 to 5.9) was described as being essential for infectivity, shedding, and transmission in wild ducks for HPAIVs [19]. However, for the efficient infection of mice and probably other mammals including humans, we found that a lower pH optimum of the HA activation might be required.

Interestingly, the mutation HA2 K58→I was first described as stabilizing the HA towards a low pH and thereby conferring resistance to the antiviral substance amantadine in a variant of the HPAI virus Rostock (H7N1) [35]. Amantadine, besides for inhibiting virus uncoating during the entry step, also prevents the release of infectious influenza virus particles comprising an HA with a polybasic cleavage site [36]. As our results indicate that the mutation HA2 K58→I increases the infectivity of H5 viruses for mice, the use of amantadine in clinics, or especially in farming, could support the appearance of H5 HPAIVs more infectious for mammals [37].

It is noteworthy that LPAIVs (H5N1) are known to have a pH range of HA activation similar to that of human influenza viruses and, therefore, are extremely stable at a low pH [18]. This property is essential for the efficient replication of viruses in the low-pH intestinal tract, which is the main site of replication for these viruses. In addition, LPAIVs circulate in wild birds and transmit through the fecal-oral route, requiring virus stability in fresh water, usually with a pH of around 6.0 [38]. In contrast, HPAIVs circulate mostly in domestic poultry and are excreted mainly from the trachea (upper respiratory tract), rather than the cloaca, and they do not persist very long in water [39]. Theoretically, stable LPAIVs might be potentially more infectious for the human upper respiratory tract. However, other factors such as the incompatibility of the avian virus polymerase complex with mammalian host factors or the temperature optimum of the polymerase activity were shown to restrict their efficient replication in mammals [40]–[45]. In this regard, it seems logical that a live influenza H5N1vaccine reassortant virus comprising the HA of the LPAIV A/Potsdam/1402-6/86 (H5N3) in combination with the polymerase complex of a human cold-adapted influenza virus was shown to replicate in the upper respiratory tract of humans [11], whereas analogous vaccine candidates comprising the HAs of VN1203 or A/Hong Kong/213/03 did not.

Intranasal vaccination with live attenuated influenza vaccines implies the infection of the nasal mucosa with the large droplet mode. Our findings suggest that an efficient intranasal vaccination may require a certain level of pH and the temperature stability of HA in order to achieve an optimal uptake of the attenuated virus by the nasal epithelial cells. In this context, any mutation destabilizing the HA appearing during vaccine construction or production can severely affect vaccine immunogenicity. In addition, since the nasal mucosa serves as the first target for large droplet settling, we believe that the HA mutations involved in modulating the pH and temperature stability, might be also important for the virus transmissibility besides the mutations regulating receptor binding specificity.

## Materials and Methods

### Cell culture

A Vero cell line was obtained from the European Collection of Cell Cultures and was adapted and further cultivated at 37°C and 5% CO_2_ in a serum-free cultivation medium (SFM; Opti-pro medium supplemented with 4 mM L-glutamine; Invitrogen). Madin-Darby canine kidney (MDCK; ATCC CCL-34) cells were cultivated at 37°C and 5% CO_2_ in DMEM medium (Invitrogen) comprising 2% Fetal Bovine Serum (FBS, Invitrogen) and 2 mM L-glutamine. Human bronchial epithelial 16HBE14o^–^ (HBE) cells (obtained from J. Seipelt, Austria) were grown in MEM (Invitrogen) supplemented with 10% FBS and 2 mM L-glutamine. For the latter the dishes, were coated with 10 µg/ml BSA (Sigma), 30 µg/ml bovine collagen type I (Promocell), and 10 µg/ml human fibronectin (BD Pharmingen) in Ham's F12 medium (HyClone). The carcinoma human alveolar basal epithelial A549 cells (obtained from J. Seipelt, Austria) were maintained in MEM supplemented with 10% FBS and 2 mM L-glutamine. Human Nasal Epithelial Cells (HNEpCs) were provided by PromoCell (Germany) and cultivated according to the manufacture's procedure.

### Viruses

The human influenza viruses A/Solomon Island/3/2006 (H1N1; NIBSC) (SL/03/06) and A/Brisbane/59/2007 (H1N1; NIBSC) (BN/59/07) were propagated in the allantoic cavity of 9- to 11-days old embryonated hen's eggs at 37°C. Allantoic fluids were collected 48 h post infection (p.i.) and clarified by centrifugation. The primary influenza isolate A/Vienna/25/07 (H3N2) (VI/25/07) (MDCK - derived, A/Wisconsin/67/05-like, HA and NA GenBank accession numbers are JF340081 and JF340082 respectively) was provided by the Austrian National Reference Centre for Influenza (Institute of Virology, Vienna, Austria). The primary influenza isolate A/St.Petersburg/14/10 (H1N1v) (SP/14/10, MDCK – derived, HA and NA GenBank accession numbers are JF340083 and JF340084) was provided by Influenza Research Institute (Saint Petersburg, Russia). Avian influenza viruses A/Vietnam/1203/04 (H5N1) (VN1203) (MDCK - derived, passage 3) and A/Thailand/01/04 (H5N1) (TH/01/04) (MDCK - derived, passage 2) were provided by the Institute for Medicinal Virology, Johann Wolfgang Goethe University Frankfurt (Frankfurt, Germany). Viral stocks were produced in MDCK cells in SFM supplemented with 5 µg/ml porcine trypsin (Sigma) cultivated at 37°C and 5% CO_2_.

The generation of the reassortant virus VN1203ΔNS1 was described previously [20]. Briefly, the 5∶3 reassortant viruses contain HA, NA, and M from VN1203 (H5N1) and the internal protein genes from the WHO influenza vaccine strain IVR-116 (H1N1). The HA polybasic cleavage site as well as the NS1 open reading frame were deleted. The virus VN1203ΔNS1-K58I differs from VN1203ΔNS1 by a single mutation in the HA2 58K→I introduced by site directed mutagenesis (Stratagene).

In addition, a corresponding couple of viruses was constructed as 5∶3 reassortants deriving the HA with a modified cleavage site as described above, the M and the NA of influenza virus VN1203 in combination with all other genes including the complete NS segment of the IVR-116 strain and named VN1203wtNS or VN1203wtNS-K58I (comprising the HA2 58K→I mutation).

For virus stocks, Vero cells were inoculated at a moi of 0.001 in SFM or MES buffer (100 mM MES, 150 mM NaCl, 0.9 mM CaCl_2_, 0.5 mM MgCl_2_; pH 5.6) and cultivated at 37°C and 5% CO_2_ in SFM supplemented with 0.25 µg/ml Amphotericin B (Amph B; Bristol-Myers Squibb) and 5 µg/ml porcine trypsin (Sigma). Infectious virus titers were determined in Vero cells by calculating the 50% tissue culture infectious doses per ml (TCID_50_/ml) according to Reed and Muench [46].

### Fusion assay

Viruses standardized to 128 HA units (HAU; determined following standard procedure) per 50 µl were diluted 1∶4 in a 1% suspension of human erythrocytes (Siemens) and incubated on ice for 1 h to allow virus binding. Then, the mixtures were pelleted at 72 g and the supernatants were removed. 100 µl of MES buffer at various pH values (from 5.0 to 6.0) were added, followed by incubating at 37°C for 1 h. After centrifugation (72 g), 50 µl of supernatant were transferred to 96-well plates and the amount of hemoglobin released by virus-cell fusion induced hemolysis was determined by the measurement of optical density at 405 nm. Reported results are the means ± standard deviations (SD) of 3 replicates at the indicated pH value.

### Effect of various pH values or lysosomotropic reagents on virus infectivity *in vitro*

Semi-confluent Vero cells were infected with virus at moi 2. Virus inoculum was prepared in MES buffer (0.25 µg/ml Amph B) at the indicated pH value or in SFM supplemented with 0.25 µg/ml Amph B and chloroquine (Sigma) or NH_4_Cl at indicated concentrations. It was applied to the cells at 37°C and 5% CO_2_ for 30 min. Then, the inoculum was replaced with SFM and 5 h p.i. cells were fixed with 4% para-formaldehyde, permeabilized with 1% Triton X100 (in PBS) and blocked (PBS + 1% BSA). Infected cells were stained with influenza anti-nucleoprotein (NP) monoclonal antibody (Millipore, 1∶5000 in PBS + 1% BSA) followed by Alexa Fluor 488 conjugated anti-mouse antibody (Invitrogen, 1∶1000 in PBS + 1% BSA). Images were taken on an Olympus CKX41 Fluorescence Microscope with connected Olympus camera system E330.

### Flow cytometry

Confluent MDCK cells were infected with virus at moi 2 in MES buffer (0.25 µg/ml Amph B) at the indicated pH values or mock-infected. 5 h p.i. cells were detached with 0.2% trypsin–EDTA (Cellgro), fixed with Fix/Perm buffer (BD Cytofix/Cytoperm Plus Kit), blocked in PBS (+1% FBS) overnight (o.n.), permeabilized and stained with the FITC-labelled mAb against NP (Imagen, UK). The percentage of NP-positive cells was determined by flow cytometry using an Epics Elite XL-MCL flow cytometer and EXPO 32 software (Coulter Immunotech, F).

### Virus growth kinetics

To determine the growth kinetics, four different cells lines (Vero, MDCK, A549, and HBE cells) were infected with VN1203wtNS or VN1203wtNS-K58I at moi 0.001 in SFM (+ 0.25 µg/ml Amph B). After incubating at 37°C and 5% CO_2_ for 30 min, the inoculum was removed and the cells were maintained at 37°C 5% CO_2_ in SFM supplemented with 4 mM L-glutamine, 0.25 µg/ml Amph B, and 5 µg/ml porcine trypsin (Sigma). Supernatants were collected 16, 24, 48, and 72 h p.i. and virus titers were determined by TCID_50_ assay on Vero cells.

### Infectivity in mice

Animal experiments were conducted in accordance with the Declaration of Helsinki and approved by National Authorities: One mouse study was approved by the Austrian regulatory authorities (MA58/000351/2009/13). The other two studies were done under the Russian Influenza Research Institutes Ethics Committee that is registered at the US Department of Health and Human Services under the IORG Number IORG0004322 Rsch inst of Influenza IRB # 1.

Groups of 6 to 8 weeks old out bred female mice were infected *i.n.* without narcosis with 20 µl of preparations of VN1203wtNS or VN1203wtNS-K58I virus at a dose of 4.5, 3.5, or 2.5 log_10_ TCID_50_/animal. 60 h p.i. nasal tissues were collected and the presence of infectious virus in 10% w/v tissue homogenates [in SFM supplemented with 1% antibiotic-mix (Invitrogen), 25 µg/ml gentamicin (Invitrogen)] was determined by TCID_50_ assay on Vero cells. Mouse infectious dose 50 (MID_50_) titers were calculated by using the method of Kerber and expressed as the TCID_50_ value corresponding to 1 MID_50_ [47].

### Histopathology

Groups of five 6 to 8 weeks old female BALB/c mice (Charles River) were intranasally (i.n.) infected under ether narcosis with 50 µl of VN1203ΔNS1 or VN1203ΔNS1-K58I viruses at a dose of 6 log_10_ TCID_50_/animal. An additional group of four mice was mock treated i.n. with 50 µl of PBS. 12 h p.i. mice were sacrificed and tissues of the respiratory tract were fixed in 7% formalin and paraffin embedded. Sections of the nasal epithelium, the trachea and lungs were prepared and influenza infected cells were detected with influenza anti-NP monoclonal antibody (1∶5000 in PBS; Millipore) followed by immunoperoxidase visualization (standard protocol). Sections were counterstained with hemalum. This animal study was approved by the Austrian regulatory authorities (MA58/000351/2009/13).

### Immunogenicity and protective efficacy in mice

Groups of 6 to 8 week old out bred female mice were immunized i.n. under narcosis once with 50 µl of VN1203ΔNS1 or VN1203ΔNS1-K58I virus at a dose of 6 log_10_ TCID_50_/animal. The control group was treated with PBS. 28 days post treatment, sera and nasal washes were taken and analyzed for the presence of vaccine strain specific antibodies by HAI assay, MNA, or ELISA. To obtain nasal secretions, salivation was induced by i.p. injection of 0.1 mg pilocarpine-HCl (Sigma). Concurrent small amounts of nasal secretions in the nostrils were immediately absorbed with the aid of sterile wicks and eluted in 50 µl of ice-cold sterile PBS for 4 h. Group-specific pools were made and stored at −20°C.

Treated animals were challenged on day 28 p.i. with 50 µl of VN1203wtNS (4 log_10_ TCID_50_/animal) virus under ether narcosis. On day 3 post challenge, 3 mice from each group were euthanized; the lung tissues were collected. The viral load was determined in a 10% w/v tissue homogenate by TCID_50_ assay on Vero cells.

### Detection of serum antibody titer by a hemagglutination inhibition assay (HAI)

Sera from the mice immunized with VN1203ΔNS1 or VN1203ΔNS1-K58I or mock-treated were diluted 1∶4 with Receptor Destroying Enzyme (RDE; Denka Seiken, Japan) and incubated at 37°C o.n. Thereafter, samples were inactivated at 56°C for 30 min and serial twofold dilutions were prepared. 25 µl/well of the standardized antigen (4 HAU/25 µl) were added. After incubating 1 h at RT, 50 µl of 1% horse erythrocytes (+ 1% BSA, Sigma) were added and the plates were incubated at RT for 1 h.

### Detection of neutralizing antibody titer by a microneutralization assay (MNA)

MNA was performed as previously described [20]. Briefly, serial twofold dilutions of RDE-pre-treated sera were combined with 50 µl of a standardized viral suspension (100 TCID_50_/50 µl) and incubated for 2 h at 37°C. Vero cells were added and incubated for 20 h, washed, and acetone fixed. An influenza A virus NP-specific monoclonal antibody conjugated with HRP (107L-Px, 4 µg/ml, Dr. E. Vareckova, Slovak Academy of Sciences) diluted in a blocking buffer (PBS containing 0.5% I-Block and 0.1% Tween-20) was added for 1 h. After adding the substrate (TMB, KPL), the average absorption at 450 nm (A_450_) was determined for the control wells of virus-infected (VC) and uninfected (CC) cells and the neutralizing endpoint (NEP) was determined by using a 50% specific signal calculation.
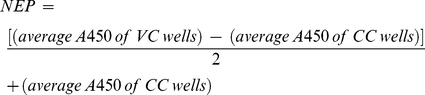



The endpoint titer was expressed as the reciprocal of the highest dilution of serum with an *A*_450_ value less than NEP.

### Detection of vaccine specific serum IgG and mucosal IgA by ELISA

A modified enzyme-linked immunosorbent assay (ELISA) protocol was performed as described previously [48], [49]. Briefly, UV-inactivated VN1203ΔNS1 virus (adjusted to 40 HAU/well in carbonate buffer; pH 9.6) was used as a coating antigen. After washing and blocking serially twofold diluted samples (serum for IgG ELISA or group specific pools of nasal washes for mucosal IgA ELISA) were added and incubated at RT for 1.5 h. H5-specific IgG or IgA antibodies were detected with goat anti-mouse IgG conjugated to HRP (0.25 µg/ml; KPL) or goat anti-mouse IgA conjugated with AP (0.25 µg/ml; Rockland Immunochemicals). Amount of IgG was determined by using TMB (KPL) substrate and level of IgA by Lumi-Phos Plus substrate (Aureon Biosystems). The cutoff values were defined as the mean value of the negative control samples plus 3 standard deviations. H5-specific IgG as well as IgA were presented in log_2_ titer.

## Supporting Information

Figure S1 **Virus stability to low pH determined by atomic force microscopy (AFM).** (A) Supported bilayer lipid membrane (sBLM) on mica: phase and topography image. Topography image of VN1203ΔNS1-K58I virus adsorbed on sBLM at pH 6.5. (B) Phase images of VN1203ΔNS1 and VN1203ΔNS1-K58I viruses on sBLM at pH 6.5–5.8–5.0. The bright regions in the phase images correspond to the liquid disordered domains in the lipid bilayer. (C) Change of the area ratio of the bright and dark regions on the phase images of the two different viruses. The error bars represent the standard deviation from five measurements.
(TIF)

Text S1 **The stability of the viruses VN1203ΔNS1 and VN1203ΔNS1-K58I to low pH analyzed by AFM.**
(DOC)
