# Supplementary figures and images for: Single HA2 Mutation Increases the Infectivity and Immunogenicity of a Live Attenuated H5N1 Intranasal Influenza Vaccine Candidate Lacking NS1
Source: PLoS One. 2011 Apr 7;6(4):e18577. doi: 10.1371/journal.pone.0018577 (PMC3072404; doi:10.1371/journal.pone.0018577)

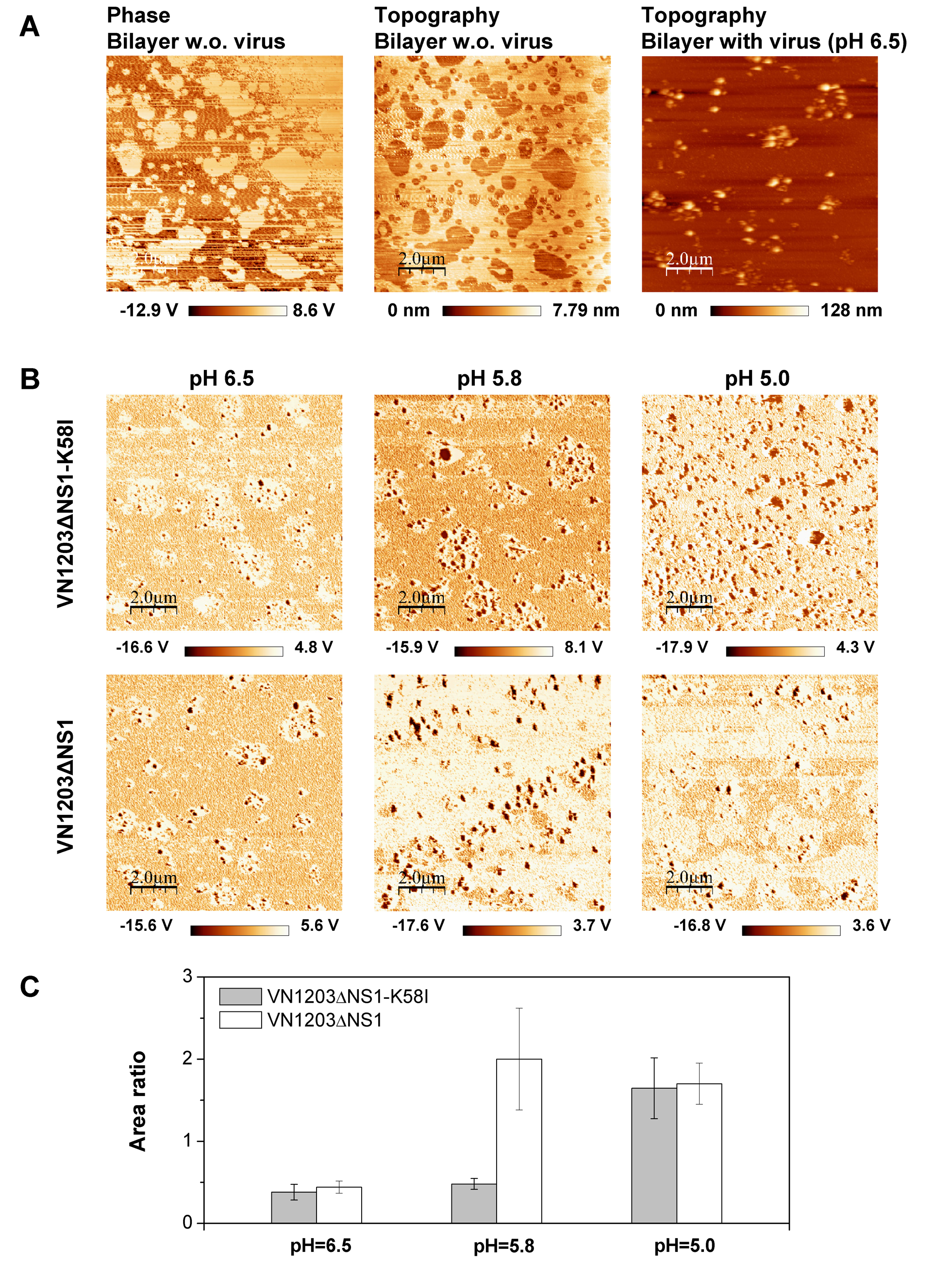

Supplement: Figure S1 — Virus stability to low pH determined by atomic force microscopy (AFM). (A) Supported bilayer lipid membrane (sBLM) on mica: phase and topography image. Topography image of VN1203ΔNS1-K58I virus adsorbed on sBLM at pH 6.5. (B) Phase images of VN1203ΔNS1 and VN1203ΔNS1-K58I viruses on sBLM at pH 6.5–5.8–5.0. The bright regions in the phase images correspond to the liquid disordered domains in the lipid bilayer. (C) Change of the area ratio of the bright and dark regions on the phase images of the two different viruses. The error bars represent the standard deviation from five measurements. (TIF) [file pone.0018577.s001.tif]
