# Supplementary material for: Single HA2 Mutation Increases the Infectivity and Immunogenicity of a Live Attenuated H5N1 Intranasal Influenza Vaccine Candidate Lacking NS1
Source: PLoS One. 2011 Apr 7;6(4):e18577. doi: 10.1371/journal.pone.0018577 (PMC3072404; doi:10.1371/journal.pone.0018577)
Supplement: Text S1 — The stability of the viruses VN1203ΔNS1 and VN1203ΔNS1-K58I to low pH analyzed by AFM. (DOC) [file pone.0018577.s002.doc]

**Supplementary information**

**The stability of the viruses VN1203ΔNS1 and VN1203ΔNS1-K58I to low pH analyzed by AFM.** Liposomes were prepared by drying a lipid solution containing dioleoylphosphatidylcholine (DOPC) and dioleoylphosphatidylethanolamine (DOPE) (Avanti Polar Lipids, USA) in a molar ratio 3:1 plus 5 mol % of GD1α (Sigma) on the bottom of a glass flask under a stream of nitrogen and resuspended in the working buffer solution (3.8 mM KH_2_PO_4_, 7.2 mM K_2_HPO_4_, 75 mM NaCl; pH 6.5) with a final lipid concentration of 1 mg/ml. After vigorous vortexing, the solution was extruded 19 times through two polycarbonate membranes (pore diameter 100 nm) to give unilamellar liposomes a relatively uniform size of approx. 100 nm in diameter. The supported bilayer lipid membrane (sBLM) was created by deposing the liposomes on freshly cleaved mica (400 µl/~2.5 cm^2^) followed by incubation for 1 h at room temperature (RT) to allow lipid bilayer formation on the support. Then, the sample was rinsed five times with the working buffer solution.

The virus preparations (~50 µg/ml) were concentrated tenfold by microfiltration through Microsep 300K filters (PallFiltron) at 40 g for 15 min. The concentrate was deposited on the sBLM and incubated at RT for 1.5 h. Then, the sample was rinsed five times with working buffer solution.

AFM measurements were performed in the magnetic AC mode (MAC) on Molecular Imaging AFM Pico SPM II, Picoplus (Agilent Technologies). The nominal spring constant of cantilevers for imaging was 0.29 N/m. All images of virus particles adsorbed on sBLM on mica were obtained in working buffer solution. During imaging, the height of the objects together with the error signal (amplitude of cantilever oscillations) and the phase lag of the cantilever oscillations were registered. Image data were processed using WSxM software [1]. To change the pH of the solution, the scanning was interrupted, the sample was taken out, and the working buffer solution was changed to the buffer with new pH value and incubated for 1 h before measurement.

The phase image was carefully leveled first, and the histogram of the image was calculated using the WSxM software [1]. From the histogram, peaks corresponding to the virus and the bright region of the lipid bilayer can be clearly distinguished. The dark region of the lipid bilayer has a wide distribution between the two peaks corresponding to the virus and the bright region of the lipid bilayer. These two peaks were fitted by single Gaussian curves and the area under the Gaussian fit of the peak corresponding to the bright region was used as the estimate of the area of the bright region of the bilayer. The area of the dark region of the bilayer was calculated by subtracting the area of the virus and of the bright bilayer region from the whole image area.

In order to analyze the stability of the viruses VN1203ΔNS1 and VN1203ΔNS1-K58I to low pH by AFM, each of the analyzed viruses was allowed to bind at pH 6.5 to the sBLM formed by the fusion of liposomes to a freshly cleaved mica surface. The molecules of GD1α ganglioside introduced to the sBLM served as a receptor for the influenza virus. In the AFM topography images of sBLM, we observed the formation of two different types of domains with height differences of approx. 1 nm (Fig. S1.A). It is known that in a three component lipid bilayer one should expect the phase separation of lipids, one being in the liquid ordered state and the other one in the liquid disordered state [2] with the former protruding out of the membrane surface [3]. Gangliosides can induce the phase separation in a lipid bilayer [2]. The suspension of virus particles was deposited on the sBLM at pH 6.5. After incubation, the AFM images of two types of virus looked similar (Fig. S1.B). We observed virus particles adsorbed on sBLM with a height ranging from 50 to 110 nm, and a diameter near 150 nm. They occupied approximately 10% of the surface area. It was interesting that the above-mentioned initial domain structure of the lipid bilayer almost disappeared. Probably the virions bound GD1α molecules so that the concentration of GD1α in the whole bilayer decreased under some critical value that led to a lower value of the line tension and the vanishing of the phase separation. The activation of fusion of the virus particles with the supported membrane at low pH values should reduce the binding forces between viral proteins and GD1α followed by the lateral spreading of GD1α molecules along the sBLM. This process should cause the reconstruction of the lipid bilayer domain structure similar to that observed before virus adsorption. Therefore, the pH was changed stepwise from 6.5 to 5.8 and to 5.0 and AFM images were taken. AFM phase images display the phase lag of cantilever oscillation relative to the driving signal, which is very sensitive to variations in the material properties of a measured sample, such as adhesion, viscoelasticity, etc. Therefore, they are especially useful for highlighting the edges of fine features, such as lipid domains.

For the VN1203ΔNS1 virus, a change in the domains formation was observed when the pH was shifted from 6.5 to 5.8 (Fig. S1.B), in turn reflecting that the virus converted the conformation at pH ≥5.8. The subsequent shift of the pH to 5.0 did not induce any additional alterations. An analogous change was observed for the mutant virus VN1203ΔNS1-K58I only at pH 5.0, indicating that this virus was more stable and required a pH <5.8 for the conformational change. In addition, the area ratio of bright regions in the phase image, corresponding to liquid disordered domains of a lipid bilayer, and the dark regions in a phase image, corresponding to the liquid ordered domains of bilayer was calculated. The data show that the area ratio increased substantially after virus conformational change for VN1203ΔNS1 at pH 5.8 and for VN1203ΔNS1-K58I at pH 5.0 (Fig. S1.C).

**Literature**

1. Horcas I, Fernandez R, Gomez-Rodriguez JM, Colchero J, Gomez-Herrero J, et al. (2007) WSXM: a software for scanning probe microscopy and a tool for nanotechnology. Rev Sci Instrum 78: 013705.

2. Akimov S.A. HEA, Bashkirov P.V., Boldyrev I.A., Mikhalyov I.I., Telford W.G., Molotkovskaya I. M. (2009) Ganglioside GM1 increases line tension at raft boundary in model membranes Biochemistry (Moscow) Supplement Series A: Membrane and Cell Biology 3: 216-222.

3. Rinia HA, Snel MM, van der Eerden JP, de Kruijff B (2001) Visualizing detergent resistant domains in model membranes with atomic force microscopy. FEBS Lett 501: 92-96.
